# Revealing potential drug targets in liver dysfunction through proteome-wide Mendelian randomization

**DOI:** 10.1097/MD.0000000000044628

**Published:** 2025-09-12

**Authors:** Xin-Mei Zhang, Tao Yang, Ying-Xin Zeng, Xin Yang, Hui-Ting Rao, Nan Hu, Guo-Dong Xia

**Affiliations:** a Department of Gastroenterology, The Affiliated Hospital of Southwest Medical University, Luzhou, China; b Health Management Center, The Affiliated Hospital of Southwest Medical University, Luzhou, China.

**Keywords:** drug targets, liver damage, Mendelian randomization, plasma proteins

## Abstract

Liver damage, ranging from mild hepatic impairment to severe liver failure, presents a significant global health burden with limited therapeutic options. Understanding the molecular underpinnings of liver damage and identifying actionable protein targets are crucial for advancing treatment strategies. We employed summary-data-based Mendelian randomization and colocalization analyses to identify plasma proteins associated with 6 liver biomarkers (aspartate transaminase, alanine transaminase, alkaline phosphatase, gamma-glutamyltransferase, total bilirubin, and direct bilirubin) by leveraging proteomic data from the deCODE cohort. Functional enrichment analyses were conducted to elucidate biological roles, and protein–protein interaction networks were constructed to explore their relationship with current drug targets for liver damage drug prediction and molecular docking studies were performed to evaluate therapeutic potential and drug–target interactions. In the summary-data-based Mendelian randomization analysis, while 21 to 106 proteins were found to be significant, only 30 were validated as potential drug targets through colocalization analysis (PP.H4 > 0.8). Enrichment analysis shows involvement in purine metabolism, nucleotide metabolism, and other pathways. Protein–protein interaction networks identified interactions with known liver damage pathways. Drug prediction highlighted potential compounds, including 7,8-benzoflavone, quercetin and tetradioxin, with strong binding affinities validated by molecular docking. This integrative study identified 30 proteins with strong evidence as potential therapeutic targets for liver damage. We provide novel insights into the molecular mechanisms of liver damage and actionable leads for drug development. Further functional and clinical validation is warranted to translate these findings into personalized therapeutic strategies.

## 1. Introduction

Liver damage, which encompasses a spectrum from mild hepatic impairment to severe liver failure, represents a substantial global health challenge, affecting hundreds of millions of individuals each year.^[1–4]^ This condition arises from various underlying causes, including viral infections such as hepatitis B and C, chronic alcohol consumption, nonalcoholic fatty liver disease (NAFLD), drug-induced liver injury, and autoimmune diseases.^[4]^ Despite significant progress in understanding the mechanisms driving liver damage, therapeutic options remain largely inadequate. Current treatments are typically limited to managing symptoms or addressing specific underlying causes, such as antiviral therapy for hepatitis or lifestyle modifications for NAFLD. However, these approaches rarely reverse existing damage or fully restore liver function, particularly in advanced stages of disease. The lack of effective regenerative therapies highlights the urgent need for innovative treatment strategies aimed at halting progression, promoting liver regeneration, or preventing complications.

Protein targets form the foundation of modern drug development, offering molecular insights into mechanisms that can be modulated to treat or prevent disease.^[5]^ Several proteins have already been identified as therapeutic targets in liver diseases, including tumor necrosis factor-alpha, interleukin-6, platelet-derived growth factor receptor, and transforming growth factor-beta receptors, which have been targeted primarily to treat hepatic fibrosis and inflammation. Traditional methods used to identify these targets typically involve biochemical assays, transcriptomics analyses, animal models, and clinical observations. Nevertheless, these conventional approaches often fail to distinguish causative targets from downstream biomarkers, limiting their translational potential. Furthermore, existing drug targets primarily alleviate symptoms rather than halt disease progression, and rarely promote hepatic regeneration or reverse liver damage. The limited clinical efficacy observed is often due to the heterogeneous etiologies underlying liver dysfunction.

Recent advances in genome-wide association studies (GWAS) have identified thousands of protein quantitative trait loci (pQTLs) associated with plasma proteins.^[6]^ These findings have opened new avenues for investigating the causal relationships between specific plasma proteins and liver function. This approach not only aids in uncovering novel protein biomarkers for liver damage but also enhances the evaluation of risk and protective factors tied to liver health.

Mendelian randomization (MR) is a well-established genetic epidemiological technique that strengthens causal inference between exposures and outcomes by using genetic variants as proxies. This approach reduces the influence of confounding variables and addresses potential reverse causation.^[7]^ The integration of GWAS data with pQTL findings through MR analysis offers a powerful framework for identifying druggable protein targets.^[8]^ Such integration enhances the efficiency of drug development by pinpointing causal relationships early in the process, reducing experimental errors, and minimizing biases.

This study leveraged the deCODE pQTL dataset as the exposure and GWAS data for 6 liver damage biomarkers as the outcomes to identify pathogenic plasma proteins and potential therapeutic targets.^[9]^ Subsequently, we further refined the results of MR analysis using colocalization methods. Furthermore, enrichment analysis was performed to better understand the biological functions and molecular interactions of the identified proteins. In addition, we evaluated the drug feasibility of these proteins and explored their relationship with current drug targets for liver damage. Drug feasibility was further supported through drug prediction and molecular docking studies, which provided insights into the pharmacological activity of these targets. These analyses not only validated the potential of the identified proteins as drug targets but also expanded the potential clinical applications of predicted therapeutic agents.

## 2. Methods

**Figure 1.**
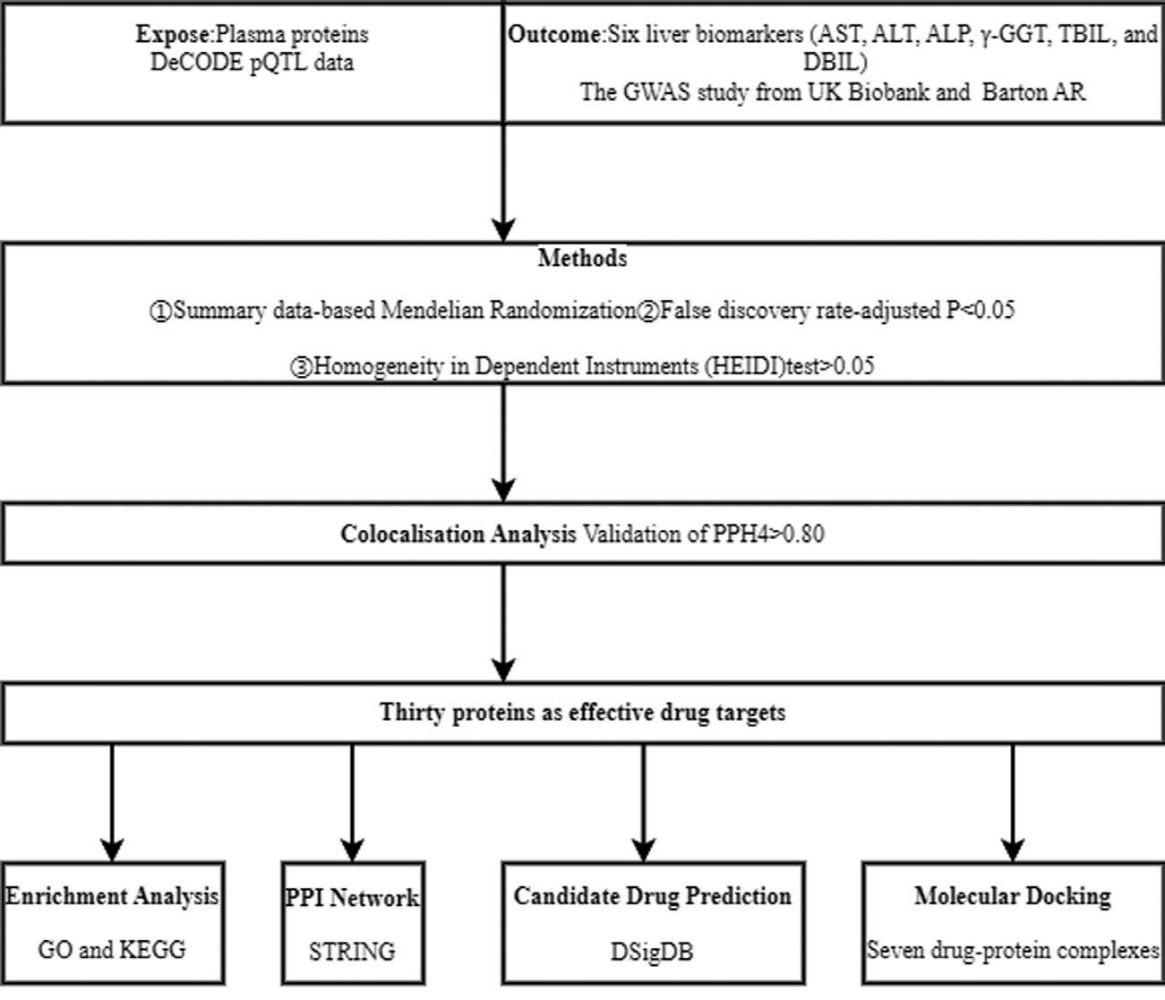
Overview of the study design.

This study utilized an integrative approach to investigate the causal association between plasma proteins and liver damage, further evaluating their biological significance and therapeutic applicability. The overall workflow, illustrated in Figure 1, highlights the systematic steps undertaken, from causal inference to drug feasibility analysis.

### 2.1. Proteomic and outcome data source

The deCODE pQTL data was analyzed using the SomaScan platform for proteomic analysis of plasma samples from 35,559 individuals of Icelandic descent, covering 4907 proteins.^[9]^ GWAS data for 6 liver damage biomarkers were sourced from various research efforts. Gamma-glutamyltransferase (γ-GGT) and total bilirubin (TBIL) measurements originated from the UK Biobank, which included 389,672 and 388,303 participants, respectively. In contrast, data on aspartate transaminase (AST; n = 436,275), alanine transaminase (ALT; n = 437,724), alkaline phosphatase (ALP; n = 437,896), and direct bilirubin (DBIL; n = 372,420) were extracted from the study conducted by Barton AR et al.^[10]^ Table S1, Supplemental Digital Content, https://links.lww.com/MD/P995 outlines the sources of all data used in this study.

### 2.2. Summary-data‑based MR analysis

The summary-data-based Mendelian randomization (SMR) method was used to examine plasma protein associations with liver damage risks, leveraging aggregated datasets. Compared to traditional MR approaches, SMR offers greater statistical power by focusing on significant cis-QTLs identified within ±1000 kb of the target gene (*P* < 5.0 × 10^‐8^).^[11]^ The heterogeneity in dependent instruments (HEIDI) test excluded pleiotropic variants (P-HEIDI < 0.05) to ensure causal validity. SMR v1.3.1 software was used for all analyses, with false discovery rates controlled using the Benjamini–Hochberg method (FDR < 0.05). Colocalization analysis confirmed associations (FDR *P* < .05, P-HEIDI > 0.05).

### 2.3. Colocalization analysis

Colocalization analysis identifies whether 2 phenotypes share a common genetic causal variant within a specific region, ruling out linkage disequilibrium or confounding factors. Using a Bayesian framework, 5 hypotheses are assessed: H0, no association with either trait; H1, associated only with the first trait; H2, associated only with the second; H3, associated with both traits but through distinct causal variants; and H4, both traits share the same causal variant.^[12]^ Posterior probabilities (PP) are calculated, with PP.H4 > 0.8 indicating significant evidence of colocalization. This approach enhances understanding of shared genetic mechanisms and strengthens causal inference in genetic studies.

### 2.4. Enrichment analysis

To evaluate the biological relevance of potential therapeutic targets, Gene Ontology (GO) and Kyoto Encyclopedia of Genes and Genomes (KEGG) pathway analyses were conducted. GO categorized genes into biological processes, molecular functions (MF), and cellular components, identifying enriched terms by comparing the target genes to the reference genome. KEGG focused on the mapping of genes to metabolic and signaling pathways. Enrichment was considered significant at *P* < .05. These analyses highlight the functional roles and pathway associations of therapeutic targets, offering insights into their relevance in disease mechanisms.

### 2.5. Protein–protein interaction (PPI) network

To explore the mechanisms of identified drug targets, a PPI network analysis was conducted using proteins identified through SMR, HEIDI, and colocalization analyses, along with established liver function drug targets. The Open Targets database (https://platform.opentargets.org/) was queried for “Decreased liver function” to identify existing therapeutic targets.^[13]^ Interactions were then analyzed using the STRING database (v12.0, https://string-db.org/) with a minimum interaction score of 0.4.^[14]^ This analysis linked novel and approved targets, revealing potential pathways and key nodes relevant to liver function improvement.

### 2.6. Candidate drug prediction

Identifying candidate drugs through drug-target interactions is essential in drug discovery. Genetic information from target proteins identified via SMR, HEIDI, and colocalization analyses was submitted to the Drug Signature Database (DSigDB) (http://dsigdb.tanlab.org/DSigDBv1.0/).^[15]^ DSigDB predicts compounds likely to interact with target genes, enabling the development of targeted therapies. This approach accelerates drug discovery and supports personalized treatment strategies by prioritizing promising candidates for further evaluation.

### 2.7. Molecular docking

Molecular docking analyses were performed to evaluate interactions between candidate drugs and their top 2 target proteins. Drug structures from PubChem and protein data from Protein Data Bank were converted to PDBQT format, with water removed and polar hydrogens added.^[16]^ Using AutoDock Vina 1.2.2, docking was conducted within a 30 × 30 × 30 Å grid (0.05 nm spacing).^[17]^ Results were visualized to assess binding interactions, supporting drug-target validation and optimization.

## 3. Result

### 3.1. MR analysis results of pQTL on liver damage

**Figure 2.**
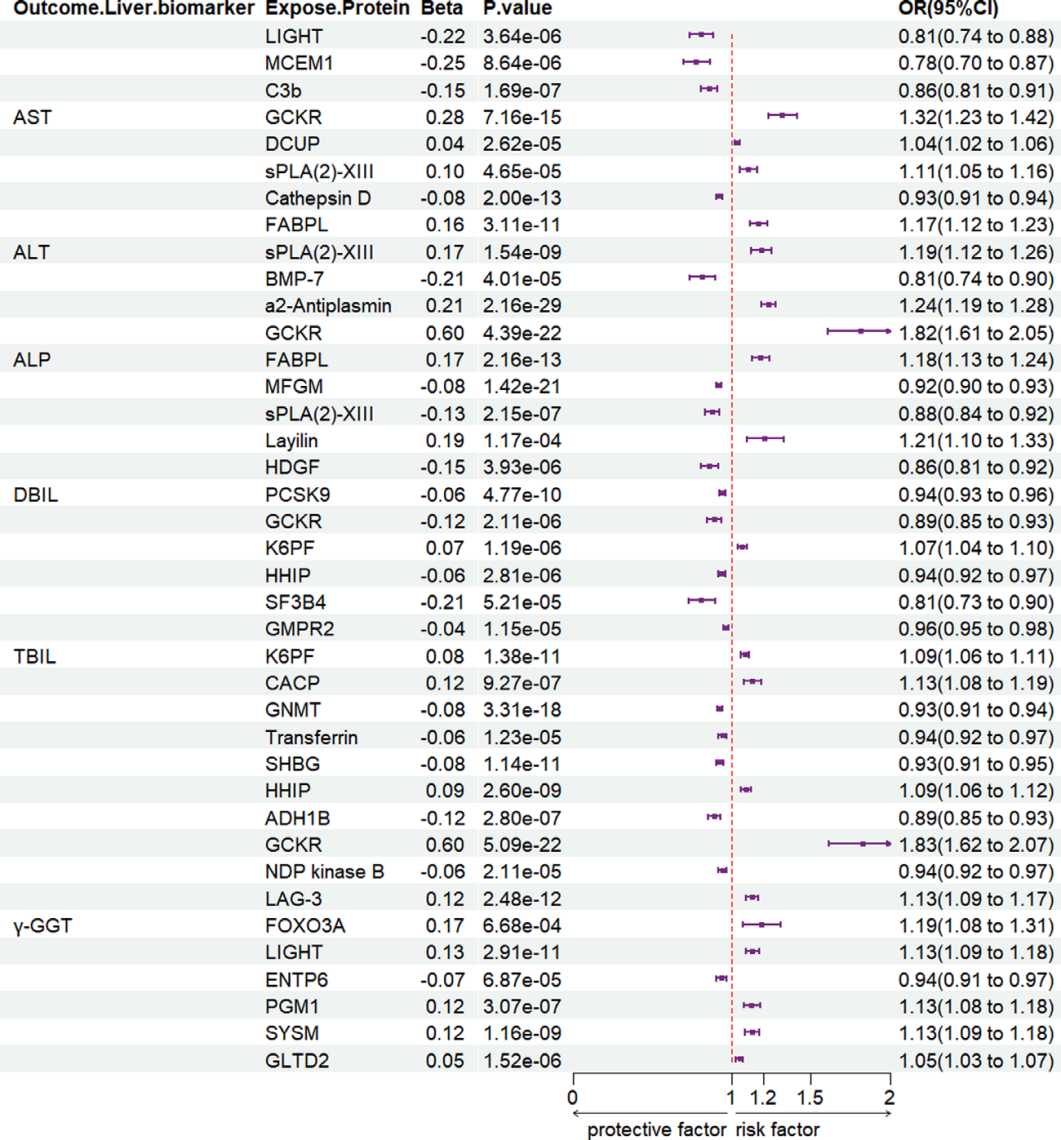
MR analysis results of pQTL on liver function. pQTL = protein quantitative trait loci.

A total of 63 to 187 proteins (refer to Table S2, Supplemental Digital Content, https://links.lww.com/MD/P995) were found to be significantly associated with liver biomarkers (AST, ALT, ALP, γ-GGT, DBIL, and TBIL) at *P* < .05, with 21 to 106 proteins remaining significant after multiple testing correction (FDR *P* < .05). As illustrated in Figure 2, colocalization analysis (PP.H4 > 0.8) identified specific associations: Elevated levels of glucokinase regulator (GCKR), DCUP (gene name not standardized, retained as DCUP), and secreted phospholipase A2 group XIII (sPLA2-XIII) were associated with an increased risk of elevated AST, whereas higher concentrations of tumor necrosis factor superfamily member 14 (LIGHT/TNFSF14), mast cell-expressed membrane protein 1, complement component 3b (C3b), and cathepsin D (CTSD) were linked to reduced AST levels. Similarly, increased concentrations of fatty acid-binding protein liver-type (FABP1/FABPL) and sPLA2-XIII were associated with a higher risk of ALT elevation, while bone morphogenetic protein 7 (BMP-7) levels were inversely associated with ALT. In the case of ALP, elevated α2-antiplasmin (SERPINF2), GCKR, FABP1/FABPL, and layilin (LAYN) levels were associated with increased risk, whereas milk-fat globule-EGF factor 8 protein/milk-fat globule membrane (MFGE8/MFGM) and sPLA2-XIII exhibited inverse associations. Higher 6-phosphofructokinase (PFKM/K6PF) levels were positively associated with increased DBIL, whereas hepatoma-derived growth factor, proprotein convertase subtilisin/kexin type 9 (PCSK9), and GCKR were inversely related to DBIL levels. Furthermore, elevated levels of 6-phosphofructokinase (PFKM/K6PF) and cartilage-associated protein (CRAT/CACP) were associated with increased TBIL, while hedgehog-interacting protein (HHIP), splicing factor 3B subunit 4 (SF3B4), guanosine monophosphate reductase 2 (GMPR2), glycine N-methyltransferase (GNMT), transferrin (TF), and sex hormone-binding globulin (SHBG) were linked to decreased TBIL levels. Finally, elevated HHIP, GCKR, lymphocyte-activation gene 3 (LAG-3/LAYN), forkhead box protein O3 (FOXO3/FOXO3A), tumor necrosis factor superfamily member 14 (LIGHT/TNFSF14), phosphoglucomutase 1 (PGM1), systromin (SYSM, if referring to a specific protein), and glycolipid transfer protein domain containing 2 (GLTPD2/GLTD2) levels were positively associated with γ-GGT elevation, whereas alcohol dehydrogenase 1B (ADH1B), nucleoside diphosphate kinase B (NDK/NME2, if referring to NDP kinase B), and ectonucleoside triphosphate diphosphohydrolase 6 (ENTPD6/ENTP6) showed inverse correlations.

### 3.2. Enrichment analysis

GO enrichment analysis revealed significant pathways for biological processes, including nucleoside diphosphate metabolism, glucose metabolism, hexose metabolism, purine metabolism, and the positive regulation of endocytosis (Figure S1, Supplemental Digital Content, https://links.lww.com/MD/P994). However, no significant pathways were identified for MF due to analytical constraints, specifically insufficient statistical power and limited functional annotation data for the MF category in the selected datasets. For cellular components, enriched structures included secretory granule cavities, cytoplasmic vesicles, the collagen-containing extracellular matrix (ECM), and endoplasmic reticulum cavities, suggesting localization within the intracellular membrane system and the ECM, with roles in secretion and transport. KEGG pathway enrichment highlighted significant genes associated with purine metabolism, glycolysis/gluconeogenesis, nucleotide metabolism, and the pentose phosphate pathway, emphasizing their importance in energy regulation and nucleic acid synthesis (Figure S1, Supplemental Digital Content, https://links.lww.com/MD/P994).

### 3.3. PPI networks

**Figure 3.**
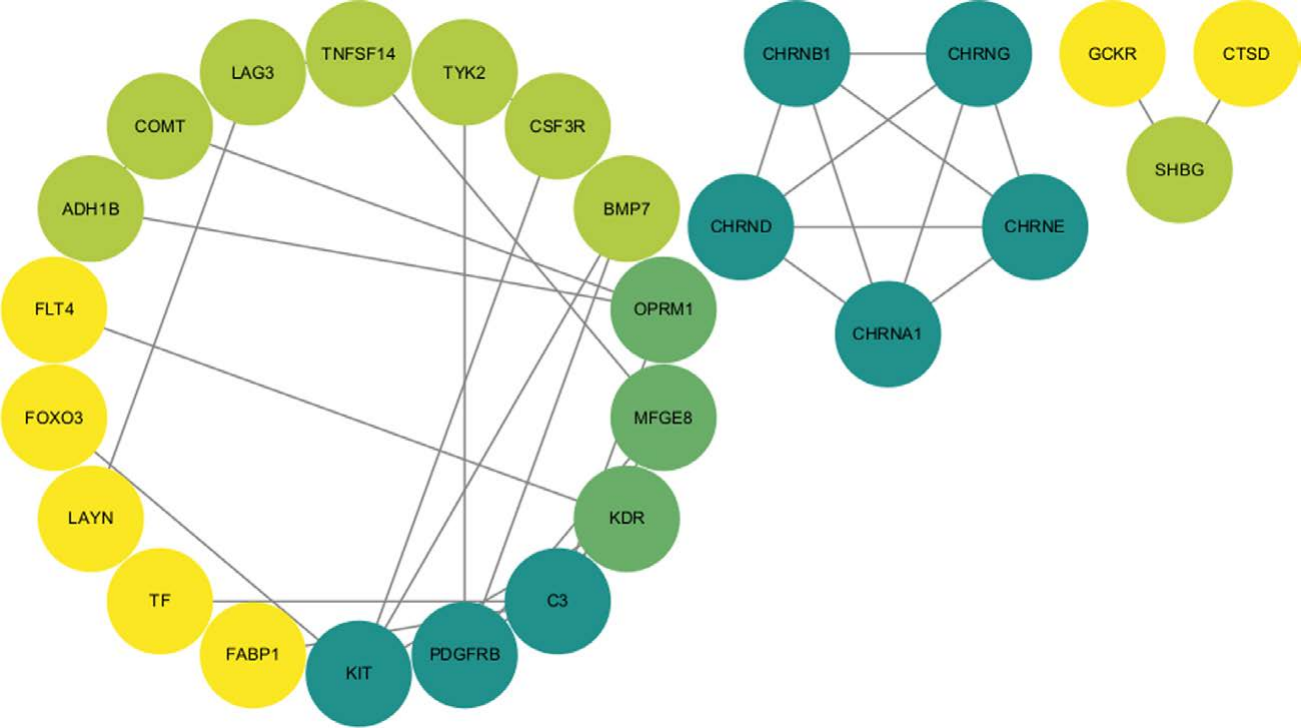
PPI network built with STRING. PPI = protein-protein interaction.

Analysis of the OpenTargets database identified 15 proteins associated with drug targets currently approved or under investigation for liver dysfunction treatment. Through PPI network analysis, 4 proteins (BMP-7, MFGM, ADH1B, and FOXO3A) were found to interact with targets of liver damage drugs (Fig. 3). BMP-7 interacts with KIT and PDGFRB, targeted by catequentinib (stem cell growth factor receptor inhibitor and platelet-derived growth factor receptor beta inhibitor). ADH1B is linked to OPRM1 and COMT, targets of methylnaltrexone (Mu opioid receptor antagonist) and tolcapone (catechol O-methyltransferase inhibitor). Additionally, MFGM interacts with PDGFRB, and FOXO3A associates with KIT.

### 3.4. Candidate drug prediction

Using the DSigDB database, this study identified 7 potential intervention compounds based on adjusted *P*-values (Table 1). The top 3 compounds, 7,8-benzoflavone (CTD 00000606), ethanol (CTD 00005337), and tetradioxin (CTD 00006848), demonstrated significant associations with genes such as FOXO3, TF, and GCKR. Due to its status as an exogenous compound with well-documented hepatotoxicity, ethanol was excluded from subsequent molecular docking analyses.

**Table 1:** Candidate drug predicted using DSigDB.

Drug names	*P*-value	Adjusted *P*-value	Genes
7,8-Benzoflavone CTD 00000606	1.00E‐05	.005	FOXO3, CTSD, PFKM, PGM1
Ethanol CTD 00005337	2.22E‐05	.005	TF, ADH1B, UROD, FOXO3, SHBG
Tetradioxin CTD 00006848	2.38E‐05	.005	GCKR, PLA2G12B, TNFSF14, ADH1B, PCSK9, LAYN, FOXO3, BMP-7, GNMT, C3, FABP1, TF, CTSD, PFKM, CRAT, PGM1
Aflatoxin B1 CTD 00007128	2.41E‐04	.037	GCKR, PLA2G12B, ADH1B, SERPINF2, PCSK9, MCEMP1, GNMT, FABP1, GLTPD2, TF, MFGE8, SHBG, PFKM
Quercetin CTD 00006679	3.10E‐04	.038	SF3B4, GCKR, PLA2G12B, SERPINF2, PCSK9, GNMT, FABP1, GLTPD2, TF, UROD, SHBG, CTSD, PGM1
Resveratrol CTD 00002483	4.02E‐04	.041	TF, ADH1B, HHIP, PCSK9, HDGF, FOXO3, MFGE8, CTSD, BMP-7
Cyclosporin A CTD 00007121	5.31E‐04	.047	GCKR, PLA2G12B, TNFSF14, ADH1B, SERPINF2, PCSK9, FOXO3, GNMT, C3, FABP1, GLTPD2, TF, HDGF, CTSD, CRAT, PGM1

### 3.5. Molecular docking

**Figure 4.**
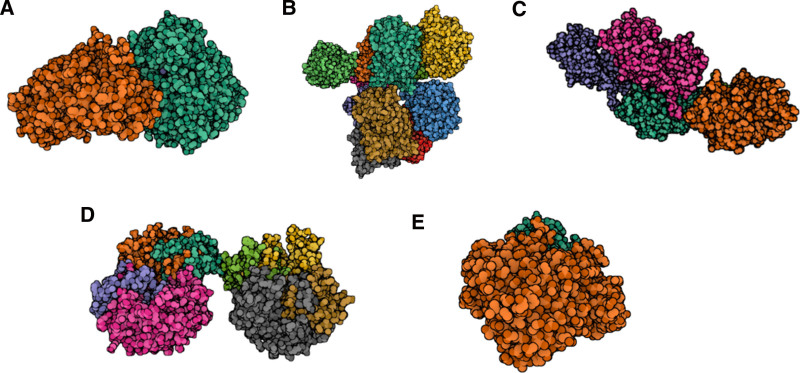
Docking results of available proteins small molecules. (A) GCKR docking with Tetradioxin. GCKR is shown in orange and green, and Tetradioxin is bound at the interface. (B) GCKR docking with Aflatoxin B1. GCKR subunits are shown in green, red, yellow, blue, gray, and purple, while Aflatoxin B1 is docked at a central pocket. (C) Transferrin docking with Resveratrol. Transferrin domains are colored pink, dark blue, green, and orange; Resveratrol is docked between the domains. (D) Cathepsin D docked with 7,8-Benzoflavone. Cathepsin D is shown in gray, orange, green, pink, and blue; the ligand is docked at the catalytic site. (E) GCKR docking with Quercetin. GCKR is represented in orange and green, and Quercetin is seen at the interface.

This study performed molecular docking using AutoDock Vina v.1.2.2 to evaluate the binding affinities of 6 candidate compounds to their respective targets, providing insights into druggability. Binding energies were calculated for all interactions, resulting in 7 successful drug–protein complexes (Fig. 4 and Table 2). Among these, the strongest interaction was between CTSD and 7,8-benzoflavone (binding energy: −10.76 kcal/mol), indicating a highly stable interaction. Additionally, GCKR exhibited stable interactions with quercetin (−9.73 kcal/mol), aflatoxin B1 (−7.75 kcal/mol), and tetradioxin (‐8.35 kcal/mol). These results highlight the therapeutic potential of the identified compounds, warranting further experimental validation.

**Table 2:** Docking results of available proteins with small molecules.

Target	PDB ID	Drug	PubChem ID	Binding energy (kcal/mol)
Cathepsin D	1LYW	7,8-Benzoflavone	11790	‐10.76
GCKR	4LY9	Quercetin	5280343	‐9.73
GCKR	4LY9	Tetradioxin	15625	‐8.35
GCKR	4LY9	Aflatoxin B1	186907	‐7.75
Transferrin	1BP5	Resveratrol	445154	‐6.6

PDB = Protein Data Bank.

## 4. Discussion

Identifying proteins that modulate liver injury is most informative when they are conceptualized not as isolated markers but as nodes within a limited set of pathogenic circuits. Joint analysis of 30 genetically supported proteins mapped onto 6 biochemical biomarkers revealed 3 interconnected axes: ECM remodeling and fibrosis, dysregulated lipid and glucose metabolism, and immune–redox homeostasis.

ECM remodeling and fibrosis. LIGHT, CTSD, BMP-7, and GNMT converge on the transforming growth factor-beta/Smad pathway that governs hepatic stellate-cell activation and type I collagen deposition.^[18–21]^ Genetic inhibition of LIGHT or CTSD, or supplementation with BMP-7 or GNMT, reduces α-smooth-muscle actin and type I collagen in preclinical models^[22–24]^; in our SMR analysis, all 4 proteins exhibited inverse associations with AST, consistent with a shared anti-fibrotic trajectory. Similarly, the whole-proteome Mendelian-randomization study by Li et al identified several ECM regulators (including CTSD) as causal drivers of NAFLD, underscoring the therapeutic promise of ECM-targeted interventions.^[25]^ Collectively, these data advocate co-targeting pro-fibrotic effectors (LIGHT, CTSD) alongside anti-fibrotic counterparts (BMP-7, GNMT) rather than relying on single-agent blockade.

Lipid and glucose dysregulation. sPLA2-XIII, GCKR, MFGM, PCSK9, and SHBG jointly determine hepatocellular lipid burden.^[26,27]^ sPLA2-XIII and the gain-of-function GCKR^rs780094 allele increase phospholipid turnover and de novo lipogenesis, respectively, correlating with elevated AST and ALT. Conversely, exogenous MFGM, PCSK9 inhibition, and higher SHBG each limit triglyceride accumulation and reduce bilirubin or transaminase concentrations in our dataset. Because these proteins operate at discrete metabolic checkpoints (membrane-phospholipid hydrolysis, glycolytic flux, LDL-receptor recycling, and sex-steroid signaling) combinatorial modulation might normalize hepatic lipid content more effectively than targeting any single node. Consistently, a genome-wide MR screen for metabolic dysfunction-associated steatotic liver disease by Ma et al validated GCKR and PCSK9 as tractable drug targets, lending empirical support to this multi-checkpoint strategy.^[28]^

Immune and redox balance. C3b, LAG-3, FOXO3A, and TF exemplify the intersection between immune surveillance and oxidative injury.^[29–33]^ Complement-driven C3b opsonization accelerates cellular-debris clearance and is inversely associated with AST, whereas the T-cell co-inhibitory receptor LAG-3 correlates positively with γ-GGT, implying sustained inflammatory activation. The stress–response transcription factor FOXO3A and the principal iron carrier TF are both negatively associated with bilirubin, suggesting that enhanced antioxidant capacity and iron sequestration attenuate haem turnover. In a proteome-wide MR study of cirrhosis, Xiao et al likewise reported protective effects of complement and iron-metabolism proteins (e.g., SERPINA1, NCAN) while systematically evaluating potential off-target consequences.^[34]^ Therapeutic regimens that bolster C3b or TF, while attenuating LAG-3-dependent cytokine release, may therefore restore immune–redox*** equilibrium.

Proteins with emerging evidence. The remaining candidates (e.g., FABPL, SF3B4, and HHIP) participate in metabolic, RNA-processing, or developmental pathways, yet their causal contributions to hepatic injury remain unresolved. These candidates warrant further investigation through functional assays and multi-omics studies to determine their mechanistic contributions and therapeutic potential. Clustering genetically validated proteins into pathway-level modules reveals synergistic intervention points (anti-fibrotic pairs [LIGHT + BMP-7], lipid-centric combinations [sPLA2-XIII + PCSK9], and immune–redox*** couplings [LAG-3 + C3b]). This network perspective converts a catalogue of individual associations into a coherent mechanistic framework capable of guiding rational polytherapy for liver dysfunction.

This study has several significant advantages, which integrates SMR and colocalization analyses to identify plasma proteins causally linked to liver dysfunction, offering actionable insights for drug development. Protein targets identified via MR exhibit high biological and therapeutic relevance, streamlining drug discovery and reducing clinical trial risks. Enrichment and PPI network analyses elucidate protein functions and regulatory pathways, while drug prediction and molecular docking evaluate pharmacological potential. Ultimately, 30 evidence-backed targets for liver dysfunction are proposed, paving the way for innovative therapeutic approaches.

However, our research also has certain limitations. Proteomic data primarily derived from European populations limits generalizability to other genetic groups, necessitating validation in diverse cohorts. Plasma protein findings may not fully reflect their roles in liver tissue, requiring liver-specific studies. Additionally, molecular docking accuracy depends on protein and ligand quality, underscoring the need for experimental and clinical validation. Despite these challenges, integrating MR with proteomics provides a powerful framework for identifying liver dysfunction targets. Addressing these limitations in future research will enhance the clinical relevance and therapeutic potential of these findings.

## 5. Conclusion

This study identified 30 potential protein targets for liver damage using SMR and colocalization analyses, validated through drug prediction and molecular docking. These findings provide a foundation for effective therapies, reducing drug development costs and advancing personalized medicine. Further experimental and clinical validation is essential to confirm the therapeutic potential of these targets and translate them into clinical applications.

## Author contributions

**Project administration:** Hui-Ting Rao.

**Resources:** Xin-Mei Zhang, Xin Yang, Nan Hu.

**Software:** Xin-Mei Zhang, Tao Yang, Ying-Xin Zeng, Nan Hu, Guo-Dong Xia.

**Supervision:** Xin-Mei Zhang, Xin Yang, Hui-Ting Rao.

**Validation:** Tao Yang, Xin Yang, Nan Hu, Guo-Dong Xia.

**Visualization:** Xin-Mei Zhang, Ying-Xin Zeng.

**Writing – original draft:** Xin-Mei Zhang, Tao Yang, Ying-Xin Zeng, Xin Yang, Hui-Ting Rao, Nan Hu, Guo-Dong Xia.

**Writing – review & editing:** Xin-Mei Zhang, Tao Yang, Ying-Xin Zeng, Xin Yang, Hui-Ting Rao, Nan Hu, Guo-Dong Xia.

## Supplementary Material


