# Supplementary figures and images for: Revealing potential drug targets in liver dysfunction through proteome-wide Mendelian randomization
Source: Medicine (Baltimore). 2025 Sep 12;104(37):e44628. doi: 10.1097/MD.0000000000044628 (PMC12440426; doi:10.1097/MD.0000000000044628)

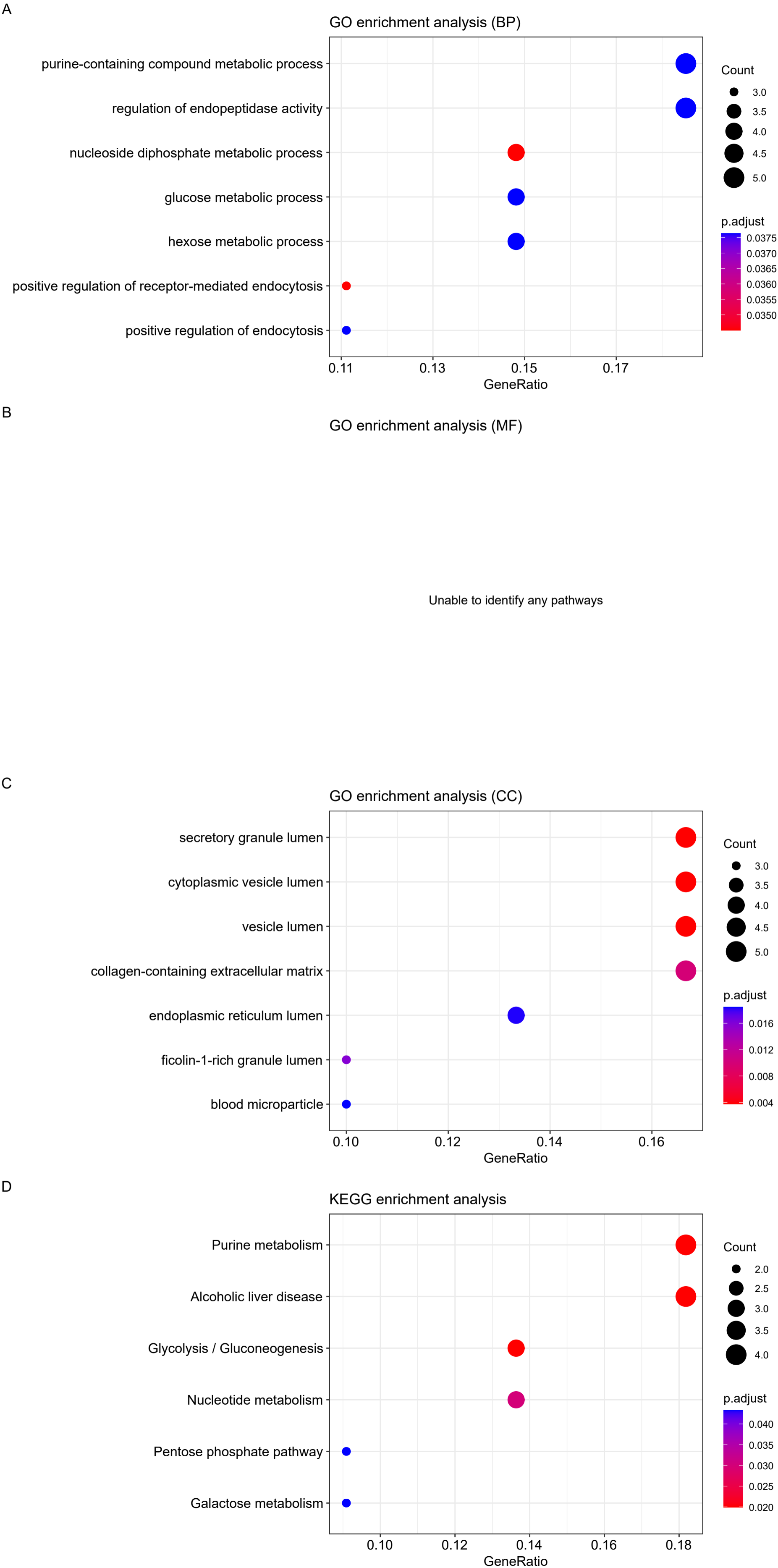

Supplement: Supplementary file 2 [file medi-104-e44628-s002.pdf]
